# Differently Charged Super-Paramagnetic Iron Oxide Nanoparticles Preferentially Induced M1-Like Phenotype of Macrophages

**DOI:** 10.3389/fbioe.2020.00537

**Published:** 2020-05-29

**Authors:** Wenyue Zhang, Shuwen Cao, Shunung Liang, Chee Hwee Tan, Baoming Luo, Xiaoding Xu, Phei Er Saw

**Affiliations:** ^1^Guangdong Provincial Key Laboratory of Malignant Tumor Epigenetics and Gene Regulation, Medical Research Center, Sun Yat-sen Memorial Hospital, Sun Yat-sen University, Guangzhou, China; ^2^Department of Ultrasound, Sun Yat-sen Memorial Hospital, Sun Yat-sen University, Guangzhou, China; ^3^The First Clinical Medical School of Guangzhou University of Chinese Medicine, Guangzhou University of Chinese Medicine, Guangzhou, China

**Keywords:** TAMs, M2-like phenotype, M1-like phenotype, tumor suppression, SPION

## Abstract

Macrophages are mainly divided into two phenotypes: M1-like (anti-tumoral, pro-inflammatory) and M2-like (pro-tumoral, anti-inflammatory). The more abundant M2-like phenotype of tumor associated macrophages (TAMs) has been associated with poor prognosis in various cancers, therefore, many studies have been carried out to modulate TAMs to change from an M2 to M1-like phenotype as an effective way to suppress tumor growth. Previous study indicated that the FDA-approved Ferumoxytol is an iron oxide nanoparticle that has intrinsic tumor inhibiting properties and is accompanied by the increased presence of the pro-inflammatory, anti-tumoral M1-like phenotype. Intrigued by this finding, we hypothesize that differently charged super-paramagnetic iron oxide nanoparticles (SPIONs) would have preferential differences in polarizing macrophages. Herein, we report that differently charged SPIONs have distinct preferences in the modulation of TAM phenotypes. Positively charged SPION (S+) had the highest cellular uptake and highest macrophage polarization effect. Interestingly, although negatively charged SPION (S−) should present charge–charge repulsion with cell membranes, they showed considerably high uptake *in vitro*, nevertheless presenting the highest cellular toxicity. Neutrally charged SPION (SN) showed minimal uptake and cellular toxicity *in vitro*. Both S+ and S− could effectively re-polarize M2-like macrophages toward M1-like macrophages *in vitro*, and significantly increased the Fenton effect and chemotaxis of macrophages. When macrophages pre-treated with these SPIONs were co-injected with tumor cells to obtain a tumor xenograft, S+ and S− treated macrophages significantly induced tumor retardation, indicating the successful repolarization of tumor macrophages by these SPIONs. Taken together, we provide an insight on the importance of SPION charge in immunomodulation of macrophages.

## Introduction

Tumor-associated macrophages (TAMs) are the most abundant immune cells in the tumor microenvironment (TME); in some solid tumors, it counts for up to 50% of the cell population (Komohara et al., 2014; Vitale et al., 2019). Macrophages could be simply classified as M1-like (pro-inflammatory) and M2-like (anti-inflammatory) phenotypes according to the expression of cytokines and chemokines (Mantovani et al., 2002). Preclinical studies have shown macrophages were largely preceding as M2-like phenotype in the TME; they accelerate tumor growth and metastasis by providing nutritional support to tumor cells, inhibiting phagocytosis, reducing activation of T cells and promoting angiogenesis (Barclay and Van den Berg, 2014; Bonapace et al., 2014; Engblom et al., 2016; Vitale et al., 2019). Thus, researchers investigated many ways to modulate TAMs, to change from M2 into M1-like phenotype, and effectively suppress tumor growth (Gunderson et al., 2016; Kaneda et al., 2016a, b; Song et al., 2016; Rodell et al., 2018), including signaling molecules, inhibitors, transcription factors, agonists, and miRNAs.

Iron oxide nanoparticles have been widely used in biomedicine, imaging and drug delivery in preclinical, and clinical settings (Anderson et al., 2019), including photodynamic therapy, anemia, magnetic drug targeting, and modulating macrophage polarization (Ma et al., 2007; Bloemen et al., 2012; Macdougall and Geisser, 2013). Ferumoxytol is an iron oxide nanoparticle that has been approved by Food and Drug Administration (FDA) for nuclear magnetic resonance imaging and iron deficiency (Lu et al., 2010). Super-paramagnetic iron oxide nanoparticles (SPIONs) are the core material in Ferumoxytol. Previous studies have shown that surface modified SPION nanoplatforms can be used for multimodal imaging and tumor therapy, such as photodynamic therapy (Kievit et al., 2012; Sivakumar et al., 2017; Zhang and Song, 2017; Zhuang et al., 2019).

Injection of Ferumoxytol could increase repolarization of TAMs and apoptosis of tumor cells, leading to the inhibition of tumor growth and metastasis (Zanganeh et al., 2016). However, whether differently charged SPIONs could have the same effect, and whether charge would influence the performance of SPIONs on tumor suppression was not clear. To clarify these questions, we synthesized three differently charged SPION (positive S+, neutral SN, and negative S−) and explored their intrinsic effect on TAMs and tumor growth.

## Materials and Methods

### Materials

Ferumoxytol (Feraheme^®^) was purchased from AMAG Pharmaceuticals Inc., Cambridge, MA, United States. SPIONs of positive, neutral, and negative change were a kind gift from Prof Dr. Morteza Mahmoudi from Harvard Medical School. All SPIONs were characterized for their sizes and charges prior to usage. Other chemicals and cell culture medium were used as purchased. Size, zeta potential and morphology were examined by dynamic light scattering (DLS) and transmission electron microscopy (TEM) respectively.

### Cell Culture

HT1080 human fibrosarcoma cells and RAW264.7 macrophages were purchased from the American Type Culture Collection (ATCC). The cells were cultured in Dulbecco’s Modified Eagle’s Medium (DMEM, Gibco, NY, United States), supplemented with 10% fetal bovine serum (FBS, Gibco) and 1% penicillin/streptomycin (Gibco).

### Cell Viability

Cells were seeded at 3000/well in a 96-well plate. After 24 h, cells were treated with respective treatment (SPIONs or Ferumoxytol) for 4 h, before washing out with phosphate buffered saline (PBS). Cells were further incubated for 48 h. Alamar Blue were added at 10 μl/well into the culture medium. After 1 h, the absorbance of the cells was determined using a Tecan UV spectrophotometer at Abs 570 nm.

### Cellular Uptake and Prussian Blue Staining

To determine the cellular uptake of all SPIONs (positive, neutral, and negatively charged) and Ferumoxytol, HT1080 cells were first seeded on a glass slide (12 × 12 mm^3^) on 24-well plate. When cells grew to ∼80% confluency, cells were treated with positively charged SPIONs (S+), Neutral-charged SPIONs (SN), negatively charged SPIONs (S−) or Ferumoxytol for 2 h at 37°C. After 2 h, cells were washed thrice with PBS before staining with Prussian Blue Kit (Abcam, Cat# ab150674; MA, United States) according to the manufacturer’s protocol.

### Transwell Assays

To evaluate the chemotactic effects of SPIONs on macrophages, HT1080 cells and RAW264.7 macrophage cells were co-cultured in dual-chamber transwell systems with 3 μm-sized microporous membranes (Corning, New York, NY, United States), which permits cell translocation between chambers. RAW264.7 cells were pre-labeled with DiD dye (Thermo Fisher Scientific, Cat# V22887; MA, United States). Briefly, 1 × 10^6^ macrophages were incubated with 5 μl of DiD dye/mL whole media SPIONs or Ferumoxytol at a concentration of 2.73 mg/mL at 37°C for 30 min. The cells were then washed thrice with PBS (pH 7.4) and collected through centrifugation (5 min, 400 RCF, 25°C) (Zanganeh et al., 2016). After washing, macrophages were plated onto transwell inserts, HT1080 cells were seeded into the bottom wells of transwell plates (Vallabani and Singh, 2018). All transwell assays were performed with DMEM, supplemented with 10% FBS and 1% penicillin/streptomycin. Cells were incubated for 24 h at 37°C in a humidified atmosphere with 5% CO_2_. Subsequently, the bottom chambers were isolated and cells were stained by 4′,6-diamidino-2-phenylindole (DAPI, Invitrogen). DiD positive macrophages that had migrated to the bottom chamber of transwell systems were counted under a Zeiss fluorescence microscope (Zeiss, Oberkochen, Germany), using DAPI and DiD channels and 40× magnification.

The same experiments were carried out to elucidate the Fenton reaction by the macrophages after treatment of various charges of SPIONs with 0.4 μm-sized microporous membranes (Corning, NY, United States). GFP-labeled HT1080 cells were shown as green while cells positive with Caspase-9 were shown as yellow punctate under 40× magnification with an Axio Zeiss microscope (Axio Observer 3.1; Zeiss, Oberkochen, Germany) and the resultant digital images were analyzed using the ImageJ (National Institute of Health, MD, United States).

### Reverse Transcription Quantitative Polymerase Chain Reaction (qRT-PCR)

To determine M1- and M2-associated gene expression *in vitro*, macrophages from the upper chamber (transwell system with 0.4 μm-sized microporous membranes and 12 h incubation time) were harvested, followed by RNA extraction with the RNeasy mini/micro kit (Qiagen, Valencia, CA, United States) according to the manufacturer’s protocol. Cell culture supernatants were also collected and either used directly or stored in −80°C until analyzed. One microgram of total RNA was reverse-transcribed into complementary DNAs with an iScript complementary cDNA synthesis kit (Bio-Rad, Hercules, CA, United States) containing a mixture of oligo (dT) and random primers. The mRNA level was determined by the comparative Ct method.

### Reactive Oxygen Species Assay

To evaluate the production of reactive oxygen species (ROS) by macrophages, RAW264.7 macrophages were co-cultured with HT1080 cells in transwell plates with 0.4 μm pore-size (12-well, Corning), which allowed free diffusion of molecules between the two chambers but not cell translocation. Culture media was retrieved from co-cultures described above and ROS levels were measured using two different ROS indicators: The hydroxyl radical was measured by incubating co-culture media with 3′-(p-hydroxyphenyl) fluorescein (HPF; Thermo Fisher Scientific, Waltham, MA, United States; Cat# H36004) at a concentration of 10 mM for 30 min at 37°C and measuring HPF fluorescence at an emission wavelength of 515 nm (excitation wavelength 490 nm). In addition, hydrogen peroxide in co-culture media was measured by incubating co-culture media with a hydrogen peroxide colorimetric detection kit for 30 min at 25°C (Enzo Life Science, Farmingdale, NY, United States; Cat# ADI907015) and absorbance was measured at 550 nm. All samples were carried out in triplicate, and the average value of ROS for each group was calculated.

### Animals

BALB/c normal mice and nude mice (female, 4–5 weeks old) were purchased from the Sun Yat-sen University Experimental Animal Center (Guangzhou, China). All *in vivo* studies were performed in accordance with the Institutional Animal Care and Use Committee at Sun Yat-sen University.

### *In vivo* Tumor Growth Analysis

To determine the effect of macrophage polarization toward tumor growth, 5 × 10^6^ HT1080 cells were mixed with either 1 × 10^6+^ of S+, SN, S− or Ferumoxytol before injecting into the right flank of Balb/c nude mice (*n* = 3). After 14 days, prior to tumor excision, Pacific-Blue Labeled Dextran^®^ (Sigma-Aldrich, Cat# FD10S; Germany) was injected to the tumor site to label TAMs. After 30 min, tumors were excised and analyzed.

### Histology

Tumor samples were embedded in Tissue-Tek opti-mum cutting temperature (OCT) compound, snap frozen in liquid nitrogen and cut into 8 μm thick frozen sections using a cryostat. Sections were fixed in ice-cold acetone for 10 min at −20°C followed by two 5 min washes in PBS and 1 h blocking in 1% bovine serum albumin (BSA)-PBS. Antibodies against CD206 (2 μg/mL, PE-conjugated, monoclonal rat anti-mouse IgG2a κ; BioLegend, San Diego, CA, United States), CD80 (4 μg/mL, Alexa Fluor 488-conjugated Armenian Hamster anti-mouse IgG, BioLegend, San Diego, CA, United States), CD11b (4 μg/mL, FITC-conjugated rat anti-mouse IgG2b κ, BD), and the corresponding isotype controls were diluted in 1% BSA-PBS to the indicated concentrations and then applied to the sections followed by overnight incubation at 4°C in the dark. Sections were washed three times with PBS for 5 min and mounted with DAPI and imaged with 40× magnification with an Axio Zeiss microscope (Axio Observer 3.1; Zeiss, Oberkochen, Germany) and the resultant digital images were analyzed using the ImageJ (National Institute of Health).

## Results

### Synthesis and Characterization of Differently Charged SPIONs

**FIGURE 1:**
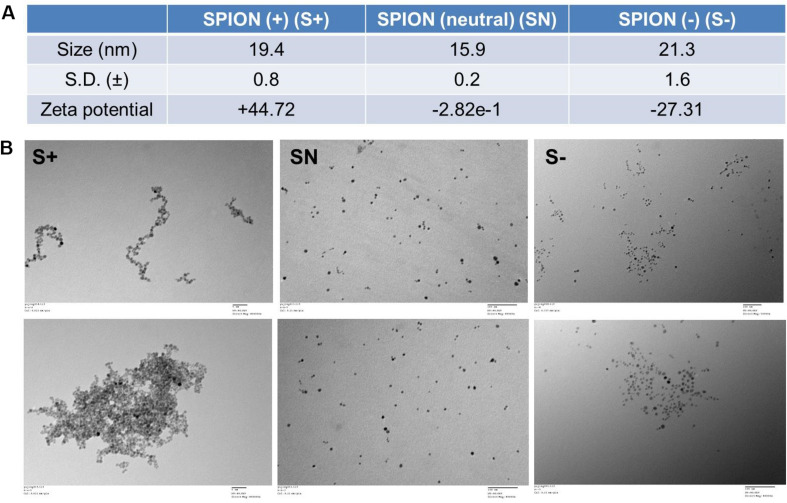
Characterization of differently charged SPIONs. **(A)** Size and zeta potential of differently charged SPIONs. **(B)** TEM images of differently charged SPIONs.

On the basis of the FDA-approved Ferumoxytol, we synthesized three differently charged SPIONs, with zeta potential of +44.72 mV (S+), −2.82e-1 mV (NS), and −27.31 mV (S−) (Figure 1A). Each particle, S+, SN, and S−, had a size of about 19.4 ± 0.8 nm, 15.9 ± 0.2 nm, and 21.3 ± 1.6 nm, respectively (Figure 1A). The morphology of differently charged SPIONs were characterized by TEM. TEM images showed that S+ aggregated easily, followed by S−, while SN due to the polyethylene glycol (PEG) coating, did not show any aggregations (Figure 1B).

### *In vitro* Uptake of SPIONs in RAW 264.7 Macrophage Cells

**FIGURE 2:**
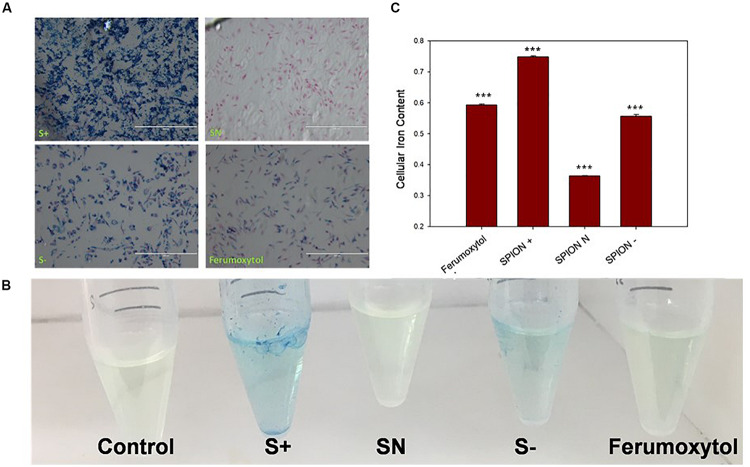
Analysis of cell uptake of Ferumoxytol and SPIONs after Prussian blue staining. Blue, Iron content; red, nucleus. **(A)** *In vitro* quantification of SPIONs after Prussian blue staining and iron assay on RAW 264.7 cells. **(B)** Iron assay of cells after treated with Ferumoxytol and SPIONs. **(C)** Quantification analysis of iron assay in **(B)**. ***P < 0.005, compared with control.

To see the *in vitro* uptake properties of each SPIONs, we carried out Prussian blue staining and iron assay on RAW 264.7 cells. As seen in Figure 2A, S+ has the highest uptake, indicated by blue staining, followed by S−, Ferumoxytol and SN. In Figures 2B,C, iron assay was performed to test the iron content with a colorimetric (593 nm) product, and the quantification of iron content is shown in Figure 2C. Compared with control, Ferumoxytol and all SPIONs groups showed significantly uptake for iron content (*P* < 0.005). Cells treated by S+ indicated the highest cellular iron content, followed by a similar amount of iron content in S− and Ferumoxytol treated cells, while the SN showed the least cellular uptake.

### Effects of Differently Charged SPIONs Toward the Repolarization of Macrophages

**FIGURE 3:**
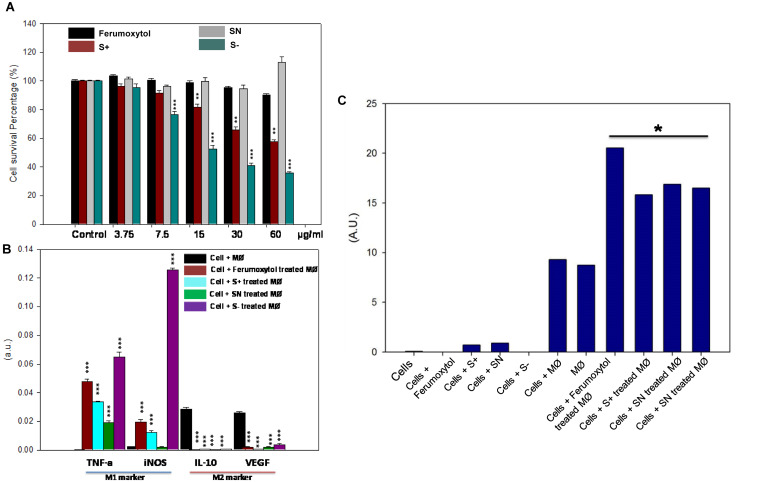
Effect of differently charged SPIONs on macrophages. **(A)** Cellular cytotoxicity of SPIONs toward RAW cells. **P < 0.01, ***P < 0.005, compared with relative control. **(B)** *In vitro* polarization of RAW cells after being treated with SPIONs. ***P < 0.005, compared with relative control (cell + macrophage group, black one). **(C)** Production of ROS after co-treatment of polarized RAW and tumor cells. *P < 0.05.

The cellular cytotoxicity of differently charged SPIONs toward RAW 264.7 cells is shown in Figure 3A. With increasing concentration, cytotoxicity of S+ and S− gradually showed significant toxicity (*P* < 0.01 for S+ group, *P* < 0.005 for S− group). S− the highest cellular toxicity compared to other groups, while SN and Ferumoxytol did not show appreciable cytotoxicity. To explore the repolarization of macrophages, RAW 264.7 cells were incubated with respective formulations for 24 h. Cells were digested and real-time RT-PCR was carried out to determine the expression of representative M1 [tumor necrosis factor-α (TNF-α), inducible nitric oxide synthase (iNOS)] and M2 [interleukin-10 (IL-10), vascular endothelial growth factor (VEGF)] marker. As seen in Figure 3B, compared with HT1080 plus macrophage only, Ferumoxytol, S+ and S− groups showed significant repolarization for macrophages (*P* < 0.005). While SN group showed significantly increase expression in TNF-α, and decrease expression in IL-10 and VEGF (*P* < 0.005). In all, S− showed the highest repolarization (from M2-like to M1-like phenotype), followed by S+ and Ferumoxytol, while the SN showed the least polarization effect.

To determine the ROS production after co-culture of polarized macrophage and tumor cells, we incubated HT1080 cells with respective treatments for 6 h, and the media was collected to explore the production of hydrogen peroxide (Figure 3C). Co-incubation of HT1080, macrophage and Ferumoxytol or differently charged SPIONs produced significant amounts of hydrogen peroxide, and the production of ROS in the Ferumoxytol treated group was significantly higher than other groups (*P* < 0.05). Treatment of (a) HT1080 + macrophage and (b) macrophage-only groups produced negligible amount of ROS, while treatment of (c) cancer cells only, (d) cancer cells + Ferumoxytol, or (e) differently charged SPIONs did not lead to significant ROS production.

**FIGURE 4:**
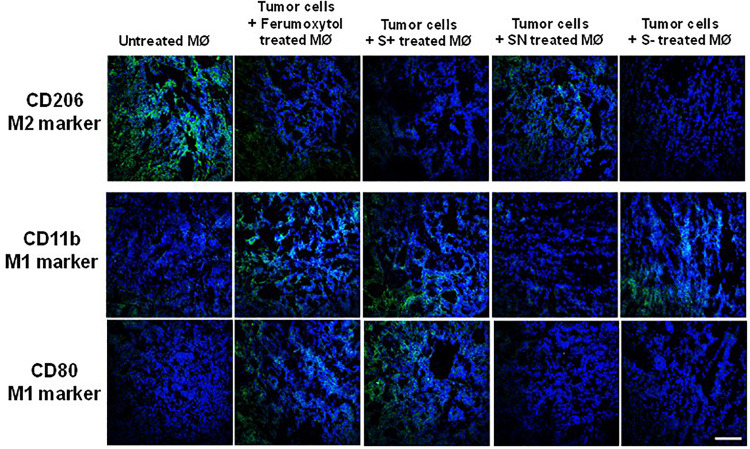
Representative immunohistochemistry staining tumor slices for CD206 (green, M2-associated marker), CD11b (green, M1-associated marker), and CD80 (green, M1-associated marker) after injection of differently treated RAW cells. Scale bar, 100 μm.

To visualize the polarization of tumor macrophages *in vivo*, we injected RAW 264.7 cells only and treated with Ferumoxytol, S+, SN, and S− intratumorally into HT1080 xenograft tumors. After 7 days, a fraction of the tumor was used for immunohistochemistry (IHC) to detect the expression of CD206 (M2-like marker), CD80, and CD11b (an M1-like marker). As evidenced by the staining in Figure 4, compared with tumor cells, Ferumoxytol, S+, and S− pre-treated groups showed higher CD11b and CD80 expression, and less CD206 expression. The SN group did not show significant changes in CD11b and CD80, but had a slight decrease of CD206 expression.

### Effect of Macrophages Treated With Differently Charged SPIONs on Tumor Cells

**FIGURE 5:**
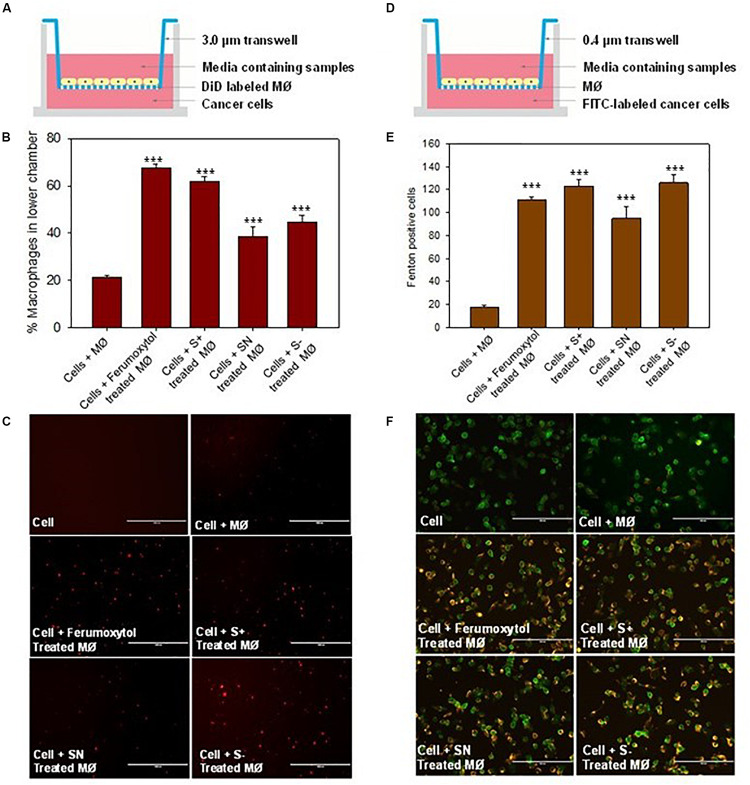
Effect of macrophages treated with differently charged SPIONs on tumor cells. **(A)** Sketches of chemotaxis effect on macrophage incubated with tumor cells. **(B)** Percentage of macrophages on lower chamber in different groups. ***P < 0.005, compared with control (cell + macrophage). **(C)** The fluorescence images of DID-labeled macrophages (red) in the lower chamber. Scale bar, 1000 μm. **(D)** Co-incubation of macrophage and tumor cells with 0.4 μm trans well. **(E)** Calculation of Fenton positive tumor cells of different groups in lower chamber. ***P < 0.005, compared with control (cell + macrophage). **(F)** Fluorescence images of Caspase 9 (yellow) expression in tumor cells (green). Scale bar, 200 μm.

To elucidate the chemotaxis effect of repolarized macrophages, we co-cultured RAW 264.7 cells on the top chamber and HT1080 cancer cells on the lower chamber of the Transwell (Figure 5A). The macrophages labeled with DiD were first treated with the respective formulations for 30 min, and then incubated on the top chamber of a 3.0 μm trans well. After 24 h, the number of DiD-positive cells were counted in the lower chamber. The number of DiD-labeled macrophages were calculated in Figures 5B,C. Compared with the untreated macrophages, the Ferumoxytol treated group had a significantly higher chemotaxis rate (67%, *P* < 0.005), followed by SN, S+, and S− and groups (53, 44, and 35% respectively, *P* < 0.005). As shown in Figure 5C, the fluorescence intensity of macrophages in the Ferumoxytol and all SPION groups was higher than HT1080 + macrophage group.

We also explored if Fenton reaction occurred when HT1080 cells co-cultured with macrophages treated with differently charged SPIONs. In this experiment, we pre-treated macrophages with Ferumoxytol and various charges of SPIONs, respective macrophages were then incubated at the upper chamber while HT1080 cells were incubated in the lower chamber of a 0.4 μm Transwell (Figure 5D). From the fluorescence images and quantitation of Fenton positive cells after 24 h, we can see regardless of SPION charge, all groups including Ferumoxytol induced significant Fenton reactions in the cancer cells, and caused high expression of caspase 9 (*P* < 0.005) (Figures 5E,F).

### Effect of SPIONs on Macrophage and Tumor Growth *in vivo*

**FIGURE 6:**
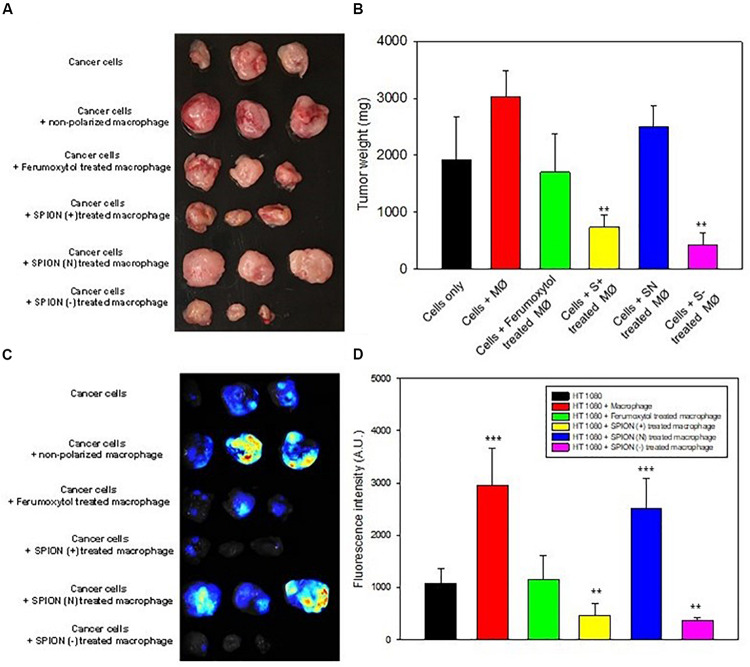
*In vivo* inhibition of tumor growth. **(A)** Images of tumor at day 14 after representative treatment. **(B)** Relative tumor sizes of **(A)**. **P < 0.01, compared with control (cancer cells). **(C)** Fluorescence images for TAMs in tumor area after different treatment as labeled by Blue Dextran^®^. **(D)** Relative fluorescence intensity of **(C)**. **P < 0.01 and ***P < 0.001, compared with control (HT1080).

To elucidate the effect of macrophage polarization toward tumor growth, we treated the RAW cells with the respective formulations and co-injected them with HT1080 cells. Images of tumors and tumor sizes are shown in Figures 6A,B, 14 days after injection. Results showed that co-injected tumors with prior treatment of Ferumoxytol, S+, and S− macrophages showed significant tumor retardation (*P* < 0.01) as compared to the treatment of tumors with non-polarized macrophages. while the SN pre-treated group exhibited no anti-tumor efficacy.

Prior to tumor excision, we injected Dextran MW10,000 tagged with Cascade blue, specially used to label TAMs. The tumor images and fluorescence intensity are shown in Figures 6C,D. As seen in both figures, the intrinsic tumor macrophages of S+ and S− groups were almost deprived compared to SN treated group or cancer cells treated with non-polarized macrophages.

## Discussion

In this manuscript, we focused on the ability of differently charged SPIONs to repolarize TAMs and suppress tumor growth. A previous study on Ferumoxytol had shown that it could induce the responses of pro-inflammatory macrophages, increase Caspase-3 expression of tumor cells, and decrease cancer progression (Zanganeh et al., 2016). Other studies on chronic venous leg ulcers, hemolytic diseases and immunotherapy for tumor have also indicated the essential role of iron in polarizing macrophage phenotypes (M1-like phenotype), and further maintaining chronic inflammation and affecting residual fibroblast (Sindrilaru et al., 2011; Vinchi et al., 2016; Costa et al., 2017). Differently charged SPIONs were able to polarize RAW 264.7 cells to an M1-like phenotype, and enhance ROS intensity in tumor cells, which could further create a Fenton reaction and cause tumor suppression. Interestingly, we found that there was efficacy difference among differently charged SPIONs, therefore, it is necessary to determine the most effective “charge-polarization” ratio for potential clinical application.

All SPIONs (S+, SN, and S−)were synthesized with a size range of 15–25 nm, a desirable size range; as they were small enough to escape from the elimination by the reticuloendothelial system; and to be easily penetrate deep into tumor (Popovic et al., 2010; Veiseh et al., 2010). The different surface charges of SPIONs could influence cellular uptake. In Prussian blue staining and iron assay of RAW 264.7 cells, S+ showed the highest uptake (*P* < 0.005), followed by S− (*P* < 0.005), while the PEG coated SN showed the least uptake. As positively charged nanoparticles were preferentially taken up by cells, while negatively charged nanoparticles could show non-specific electrostatic interactions with proteins on the cell membrane; it is not surprising that both S+ and S− showed significant cellular uptake.

Our data demonstrated both S+ and S− were able to repolarize macrophages, as evidenced by the higher expression of TNF-α, iNOS, CD11b, and CD80, and lower expression of IL10, VEGF and CD206, which were characteristic hallmarks of M1 and M2-like macrophages, respectively. Previous *in vivo* studies on SPIONs confirmed they were ingested by macrophages and would be degraded in lysosomes, causing iron accumulation (Weinstein et al., 2010). The accumulated iron could up-regulate ferritin and cathepsin L levels in macrophages, and alter M2-like macrophages to an M1-like phenotype, with an increased expression of CD86 and TNFα (Laskar et al., 2013).

Macrophage repolarization was also evidenced by the co-culture of tumor cells with pre-treated macrophages (S+, SN, S−). Co-culture of S+ and S− pre-treated macrophages with HT1080 triggered a high induction of ROS which then triggered an increased expression of Caspase 9, leading to apoptosis. Repolarized macrophages were reported to produce superoxide anions, oxygen radicals and nitrogen radicals (Sindrilaru et al., 2011). As the macrophages were treated with iron oxide nanoparticles, they produced ROS and could further react with iron by Fenton reaction, starting the programmed cell death of tumor cells by Caspase-dependent apoptosis (Zanganeh et al., 2016). Combined with the proinflammatory cytokines released by repolarized macrophages, the killing activities for tumor cells could be effectively enhanced.

S+ and S− pre-treated macrophage proved to have the highest tumor inhibition ability (Figures 6A,B). Ferumoxytol-treated macrophage also indicated a significant inhibition, while SN-pretreated macrophage did not show appreciable tumor retardation. And the number of TAMs in the tumor area was consistent with the efficiency of tumor retardation in Ferumoxytol and differently charged SPIONs groups. Our results demonstrate that both positive and negatively charged SPIONs had higher repolarization abilities *in vitro* and *in vivo*. Previous studies on immune cells had proved that repolarized macrophages could enhance the inflammatory reaction by cross regulating some signal pathways such as toll-like receptors (TLR) and tumor necrosis factor (TNF) on STAT3, and finally activating the essential pathway of nuclear factor kappa B (NF-κB) to trigger inflammatory reaction (Stark and Darnell, 2012; Glass and Natoli, 2016; Murray, 2017). In a nutshell, M1-like macrophages could produce proinflammatory cytokines like TNF-α and iNOS, and the newly recruited monocytes might also be triggered to modulate to an M1-like phenotype by iron stored in tumor area, forming the cycle for further enhancement of their antitumor effect.

## Conclusion

In conclusion, our results confirmed the importance of surface charge for SPIONs in phenotype polarization of macrophage. Compared to SN, S+, and S− could significantly repolarize TAMs and suppress tumor growth. However, considering the toxicity of SPIONs in high concentration, S+ may have greater potential for further clinical anti-tumoral application.

## Data Availability Statement

All datasets presented in this study are included in the article/supplementary material.

## Ethics Statement

The animal study was reviewed and approved by the Sun Yat-sen Memorial Hospital.

## Author Contributions

PS and XX oversaw all the experiments. WZ and SC carried out all the experiments. SL and CT did all the analysis. BL revised the manuscript.

## Conflict of Interest

The authors declare that the research was conducted in the absence of any commercial or financial relationships that could be construed as a potential conflict of interest.
